# Dual-Wavelength Metalens Design for Compact LWIR and MWIR Imaging Systems

**DOI:** 10.3390/s26051536

**Published:** 2026-02-28

**Authors:** Ting Liu, Kun Zheng, Shibin Jiang, Zhirui Zeng, Guanxing Zang, Wei Huang, Weiming Zhu

**Affiliations:** 1 School of Optoelectronic Science and Engineering, University of Electronic Science and Technology of China, Chengdu 610054, China; 202421050409@std.uestc.edu.cn (T.L.); 202411050925@std.uestc.edu.cn (K.Z.); 202211050843@std.uestc.edu.cn (S.J.); 202311050808@std.uestc.edu.cn (Z.Z.); 2 Hisense Laser Display Co., Ltd., 399 Songling Road, Qingdao 266555, China; 3 Key Laboratory of Multifunctional Nanomaterials and Smart Systems, Key Laboratory of Semiconductor Display Materials and Chips, Suzhou Institute of Nano-Tech and Nano-Bionics (SINANO), Chinese Academy of Sciences (CAS), Suzhou 215123, China

**Keywords:** dual-wavelength metalens, LWIR and MWIR optics, thermal imaging, inverse design

## Abstract

Multispectral infrared imaging systems that simultaneously operate in the long-wave infrared (LWIR) and mid-wave infrared (MWIR) bands offer significant advantages for target detection and recognition. However, conventional infrared optical systems rely on bulky multi-element lens assemblies to accommodate incident wavelengths of LWIR and MWIR bands, making it challenging for compact thermal optics design. Here, we propose and experimentally demonstrate an inverse designed infrared metalens capable of simultaneously focusing LWIR and MWIR radiation at wavelengths of 9.5 μm and 4.75 μm with a focal length variation of 1%. In the experiment, the proposed metalens with detector enables a dual-wavelength thermal imaging with a compact size (26 × 26 × 18 mm^3^) and a light weight (19 g). This work establishes a robust and scalable inverse design strategy for dual-wavelength infrared metalenses and provides a promising route toward compact, integrated, and multifunctional infrared imaging lens.

## 1. Introduction

Thermal imaging technology has found extensive application in domains such as military surveillance, medical diagnostics, and industrial inspection, owing to its passive operational characteristics, versatility in temperature measurement, and capacity for material discrimination [1,2,3,4,5]. Distinct advantages are associated with long-wave infrared (LWIR) and mid-wave infrared (MWIR) radiation under varying environmental conditions. Specifically, LWIR demonstrates superior performance in atmospheres containing elevated concentrations of aerosols, smoke, or dust, whereas MWIR exhibits enhanced suitability for transmission in high-humidity environments [6,7]. From a radiative perspective, large structures such as ships and aircraft predominantly emit thermal signatures in the LWIR spectrum, while engines and exhaust plumes are more pronounced sources within the MWIR range. The integration of LWIR and MWIR imaging through dual-channel acquisition enables the capture of complementary radiative characteristics [8,9], thereby facilitating more comprehensive scene representation, improving target recognition accuracy, and enhancing the overall reliability of thermal imaging systems.

Conventional infrared imaging systems typically rely on cascaded refractive or diffractive lens assemblies composed of multiple optical lens with varying materials and geometries [10,11,12,13]. The dispersion properties of optical materials exhibit pronounced differences between the LWIR and MWIR spectral bands, necessitating the use of multiple refractive lenses to correct chromatic aberrations. In addition, phase accumulation in traditional optical systems depends on bulky refractive or diffractive components, leading to inherent drawbacks such as large volume, heavy weight, and complex optical design. In the field of thermal imaging, although conventional optical solutions remain dominant, emerging technologies such as mid-infrared photonic lanterns based on fluoride glass have recently attracted growing research attention due to their significant potential [14,15,16]. These limitations severely restrict the deployment of infrared imaging systems in emerging application scenarios that demand lightweight, compact, and highly integrated solutions, such as unmanned aerial vehicles, wearable devices, and multispectral cooperative sensing platforms [17,18,19,20,21].

Unlike conventional lenses that rely on phase accumulation during propagation, metalenses achieve abrupt phase modulation within a subwavelength-thick layer, thereby replacing bulky multi-lens assemblies and significantly reducing system size and weight [22,23]. Numerous studies have been devoted to the development of metalenses for infrared optical applications, including achromatic beam shaping, wide-angle focusing, and beam steering [24,25,26,27,28]. Proposals for metalenses operating in either the LWIR or MWIR spectral bands have demonstrated the feasibility of dual-wavelength thermal imaging using silicon-based platforms [29,30,31,32,33,34]. Recent investigations have reported a dual-layer fabricated metalens capable of functioning across both LWIR and MWIR regimes, though with limited correction of achromatic aberrations and reduced efficiency [35]. Achieving dual-wavelength thermal imaging through simplified fabrication strategies remains a substantial challenge. This difficulty stems from the inherent trade-off between unit cell design and phase profile optimization across both wavelengths, which imposes fundamental constraints on the performance of metalenses operating simultaneously in dual spectral bands.

This work presents an inverse designed metalens that enables dual-wavelength functionality within the LWIR and MWIR spectral bands, fabricated via a single-step silicon etching process. In contrast to conventional forward-design methodologies, the proposed inverse design approach simultaneously optimizes focusing performance at both wavelengths. By explicitly incorporating the optical characteristics derived from the unit cell database, the optimization process is rigorously constrained through a loss function that quantitatively evaluates the overall performance of the dual-wavelength metalens. The framework incorporates the contribution of each unit cell to the overall lens performance, thereby mitigating performance discrepancies and ensuring balanced focusing quality across both the LWIR and MWIR regimes. This work establishes a robust and scalable inverse design paradigm for dual-wavelength metalenses, offering a promising pathway toward compact, integrated, and multifunctional optical systems.

## 2. Design Methodology of the Dual-Wavelength Metalens

**Figure 1:**
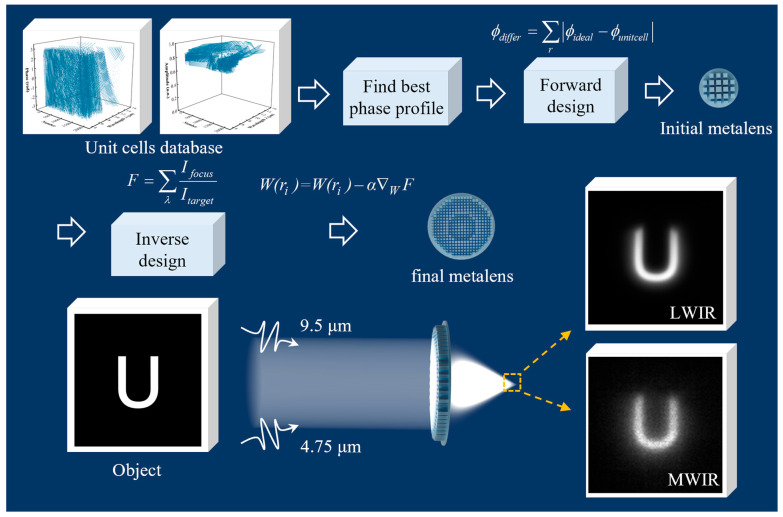
Conceptual diagram of a dual-wavelength confocal metalens. A simplified design process for the dual-wavelength metalens, including the selection of the optimized parameters, forward design for the central region and inverse design for the selection of the unit cells.

We propose an inverse design methodology for a metalens capable of simultaneously detecting information at both LWIR and MWIR wavelengths, as schematically illustrated in Figure 1. The incident light with two wavelengths of 9.5 μm and 4.75 μm are both focused onto a single detector plane by the dual-wavelength metalens. Despite the harmonic (frequency-doubling) relationship between the two selected wavelengths, the unit cells exhibit strong phase dispersion. This renders it difficult for the conventional look-up-table approach to simultaneously match the phase of the unit cells to the ideal phase profiles at both wavelengths, as described by Equation (1),where ϕ is the ideal hyperbolic phase profile, λ is the wavelength of the incident light, f is the focal length, r is the radius of the lens. Therefore, this work adopts an inverse design method with focusing efficiency as the loss function, which can more effectively optimize the spatial arrangement of the unit cells on the metalens [36].

(1) $$\phi =-\frac{2\pi }{\lambda }\left(\sqrt{r^{2}+f^{2}}-f\right)$$

Here, we adopt a ring-by-ring optimization strategy for the design of the metalens owing to the circular symmetry of the phase profile. First, based on the dispersive phase characteristics extracted from the unit cell database, two target phase profiles corresponding to the selected wavelengths are determined. These phase profiles serve as the initial input for the inverse design of the metalens, which varies with the choices of the unit cells. Subsequently, an appropriate loss function is formulated, and an inverse design optimization is performed to complete the metalens design. Finally, both the numerical and experimental characterizations are conducted to verify the performances of the dual-wavelength metalens.

**Figure 2:**
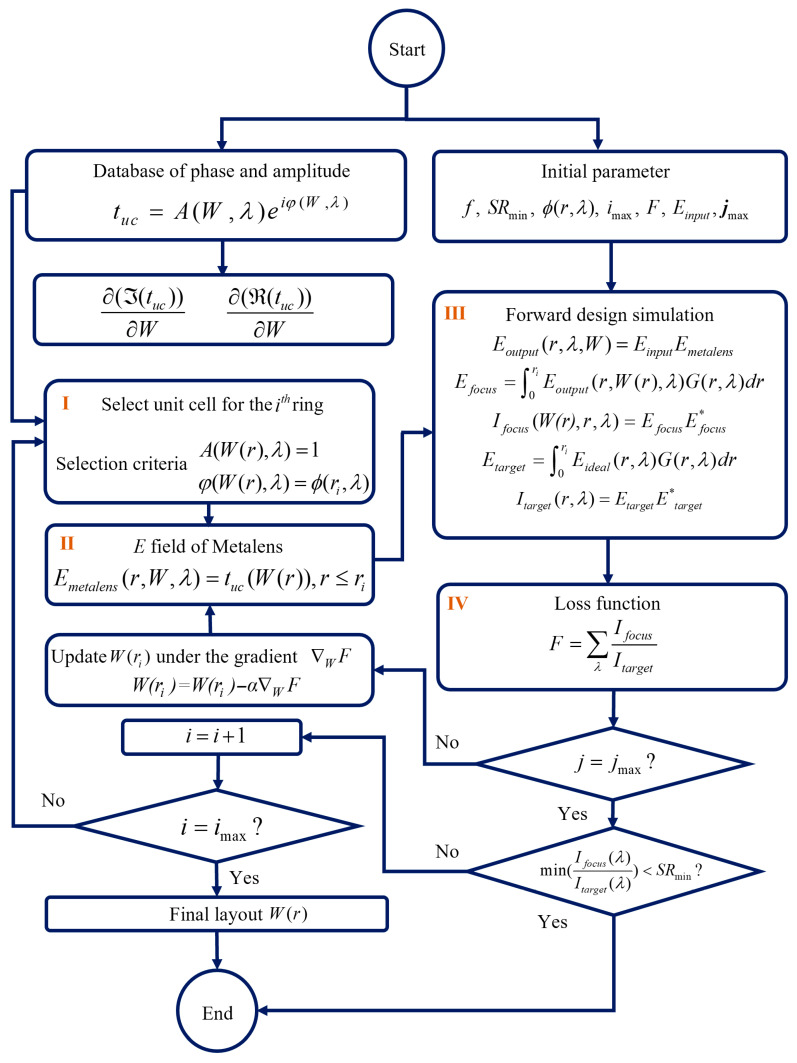
The flowchart of the inverse design for the dual-wavelength metalens.

The overall design workflow of the inverse design is shown in Figure 2, which optimizes the choices of the unit cells ring-by-ring with four core steps. First, A hyperbolic phase profile is initially adopted as the ideal phase function. Ten-unit cells are first selected from the database based on the initial phase profiles using the look-up-table method. The reason for selecting ten-unit cells using the look-up-table method is that the subsequent inverse design step requires evaluating the optical field behind the unit cells as the optimization criterion. If too few unit cells are used in the initial arrangement, the calculated focal field behind the lens may suffer from large errors, which can adversely affect the stability and accuracy of the inverse design process.

The results of the inverse design algorithm critically depend on the selection of the initial ideal phase profile. Therefore, the ideal phase profiles for the two wavelengths should be appropriately modified to facilitate continuous unit cell selection. The modulated phase profile $\phi_{m}$ is expressed as

(2) $$\phi_{m}=-\frac{2\pi \omega }{c}\left(\sqrt{r^{2}+f^{2}}-f\right)-{\left(\frac{\omega }{c}\right)}^{g}l+\phi_{phase\_add}$$

where ω is the frequency of the incident light, c is the speed of the light in vacuum, g and l are scanning parameters. The latter two terms serve as phase modulation terms. Specifically, the first modulation term ${\left(\frac{\omega }{c}\right)}^{g}l$ compensates for the wavelength-dependent phase mismatch induced by dispersion, while the second term $\phi_{phase\_add}$ introduces a phase shift without altering the phase gradient. A rough parameter sweeps over ${\left(\frac{\omega }{c}\right)}^{g}l$ and $\phi_{phase\_add}$ is first performed to align the phase profiles into suitable regions of the phase space. Second, for each sweep condition, unit cells are ranked by minimizing the phase difference with equal weighting applied to both 9.5 μm and 4.75 μm. The global phase difference $\phi_{differ}$ is defined as

(3) $$\phi_{differ}=\sum_{r}\left|\phi_{phase\_ideal}-\phi_{unitcell}\right|$$

where $\phi_{unitcell}$ denotes the phase shift in the available unit cells in the database, corresponding to the configuration that minimizes the $\phi_{differ}$. When the summed absolute phase differences across all radial positions reaches its minimum, the rough sweep is completed. Following the conventional look-up-table method, ten candidate unit cells are first selected from the database. Then the electric field $E_{focus}$ and the optical intensity $I_{focus}$ at the focal point is calculated. The electric field $E_{focus}$ and optical intensity $I_{focus}$ at the focus plane, as a function of radial *r*, wavelength λ, which can be expressed as

(4) $$E_{focus}=\int_{0}^{r_{i}}E_{metalens}\left(r,\lambda ,W\left(r\right)\right)G\left(r,\lambda \right)dr$$

(5) $$I_{focus}=E_{focus}E_{focus}^{*}$$

where *W*(*r*) denotes the width of the unit cell at radius *r*. $G\left(r,\lambda \right)=-\frac{ik}{4}H_{1}^{\left(1\right)}\left(kl\right){\vec{n}}_{i}\cdot \frac{\vec{l}}{\left|l\right|}$ and $\vec{l}$ is the displacement from metalens unit cell to the focal point on the detector plane, *k* is the wavevector of the incidence, *H*_1_ is the Hankel function and $\vec{n_{i}}$ is the unit vector of the metalens’ normal.

The subsequent unit cell arrangement is obtained using inverse design. For the $i^{th}$ ring (i≥11), all candidate unit cells in the database are sequentially placed at the radial position, and corresponding focal optical intensity is evaluated. The ratio between the calculated intensity $I_{focus}$ and the theoretical intensity $I_{ideal}$ calculated by ideal phase profile is used to determine the optimal unit cell at each position. The loss function *F* is defined as

(6) $$F=\sum_{\lambda }\frac{I_{focus}}{I_{ideal}}$$

We implement a gradient-based continuous optimization framework by fitting the unit cell width *W* to the complex transmitted field. In the optimization process, equal weights are assigned to the two wavelengths of 9.5 μm and 4.75 μm. The inverse design optimization is allowed to proceed while progressively extending the unit cell arrangement toward larger radial position until the maximum achievable diameter is reached as the inherent trade-off between chromatic dispersion and lens diameter. As indicated in Steps III and IV of Figure 2, the loss function *F* is a function of *W*. Therefore, $\nabla_{W}F$ the gradient of *F* with respect to *W* can be calculated. Using this gradient information, $W(r_{i})=W(r_{i})-\alpha \nabla_{W}F$ is iteratively updated via gradient descent to progressively approach the optimal value, where α denotes the learning rate used in the optimization. The optimization process was finalized upon the selection of the design parameter *W*(*r*) for the outermost ring.

To ensure that suitable unit cells can be found from the database, The outward placement of unit cell is terminated when the loss function exceeds the predefined threshold of 0.6.

## 3. Numerical Simulations

**Figure 3:**
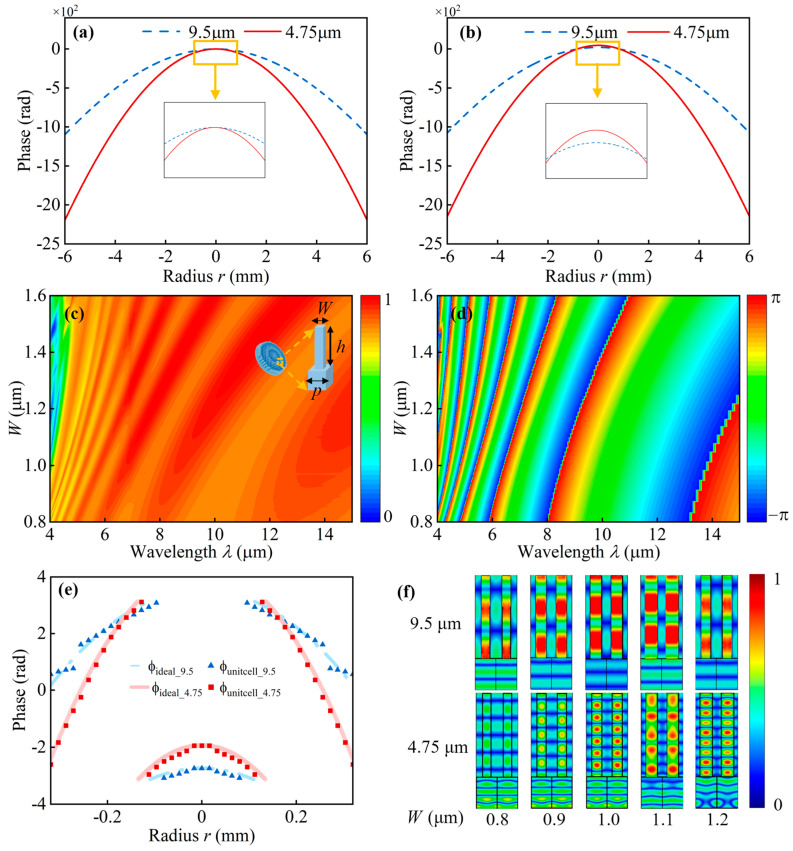
Numerical simulations on the initial phase profiles and unit cells. (**a**,**b**) show the unwrapped ideal phase and modulated phase profiles used for the inverse design, the yellow box shows a magnified view of the phase; (**c**,**d**) show the transmitted amplitude and phase of the unit cells as functions of the side wall length *W* and incident wavelength *λ*; the yellow dash line shows a magnified view of the unit cell; (**e**) Phase distribution of the metalens radius from −0.32 mm to 0.32 mm; (**f**) Electric field distributions of the unit cells with different *W* illuminated by incidences with wavelengths of 9.5 μm and 4.75 μm.

The ideal hyperbolic phase profiles and the corresponding modulated phase profiles are depicted in Figure 3a and Figure 3b, respectively. The blue-dashed lines and red-solid lines represent the phase profiles of the metalens with an incident wavelength of 9.5 μm and 4.75 μm, respectively. The selected unit cells should achieve 2π phase coverage for both wavelengths, given the minimum allowable fabrication linewidth (0.5 μm). Additionally, the unit cell should ensure polarization insensitivity.

We constructed a unit cell database for the metalens using the commercial software CST (2023) with finite-difference time-domain (FDTD) simulations. To ensure polarization insensitivity, the unit cells were designed with symmetric square pillar geometries. The unit cells and the substrate were made of silicon. Periodic boundary conditions were applied in the X and Y directions, while open boundary conditions were used in the Z direction. A normally incident linearly polarized plane wave was used as the excitation. Additionally, the performance of the metalens was verified through full-wave simulations based on the Kirchhoff diffraction formula, the results of which are essentially consistent with those obtained from FDTD based full-wave simulations [37].

The unit cells of the proposed metalens are composed of square silicon pillars with a period of 2 μm and a height of 8 μm, with side lengths varying from 0.8 to 1.6 μm. Figure 3c,d present contour maps illustrating the amplitude and phase of the unit cells as functions of pillars’ length *W* across different wavelengths, demonstrating complete 2π phase coverage. Furthermore, the all-silicon configuration is inherently compatible with conventional semiconductor fabrication techniques and can be implemented through a single-step silicon etching process.

Figure 3e shows the phase profiles of the central part of the dual-wavelength metalens designed by the inversed method. The blue triangle and red rectangle symbols represent the phases of selected unit cells with an incident wavelength of 9.5 μm and 4.75 μm, respectively. The blue-dash-dotted line and red-solid line represent the modulated phase profiles with the incident wavelength of 9.5 μm and 4.75 μm, respectively. The initial ten-unit cells are selected by using the looked-up table method based on the modulated phase profiles. The rest unit cells are selected via the inverse design method because it is not possible to find unit cells matching the phase profiles for both wavelengths within a limited unit cell database. The required phase responses of certain unit cell vary dramatically for the two wavelengths according to the modulated phase profile. For instance, at *r* = 0.16 mm, the selected unit cell shows identical phase responses at both 9.5 μm and 4.75 μm, while at *r* = 0.32 mm, the phase difference between the two wavelengths becomes relatively large, approaching π.

The electric-field distributions of the selected unit cells are presented in Figure 3f. The upper and lower rows correspond to incident wavelengths of 9.5 μm and 4.75 μm, respectively. Both wavelengths excite propagation modes within the unit cells. However, the propagation constants associated with the 4.75 μm incidence are greater than those observed at 9.5 μm. Moreover, the propagation constants at 4.75 μm exhibit heightened sensitivity to geometric variations in the unit cells, specifically the side length *W* of the silicon pillars. As *W* varies from 0.8 μm to 1.2 μm, the phase response difference between the two wavelengths spans a range from 4 π to 8 π. Consequently, by appropriately selecting *W*, a wrapped-phase difference within the range of 0 π to 2 π can be achieved, as summarized in Table 1.

**Figure 4:**
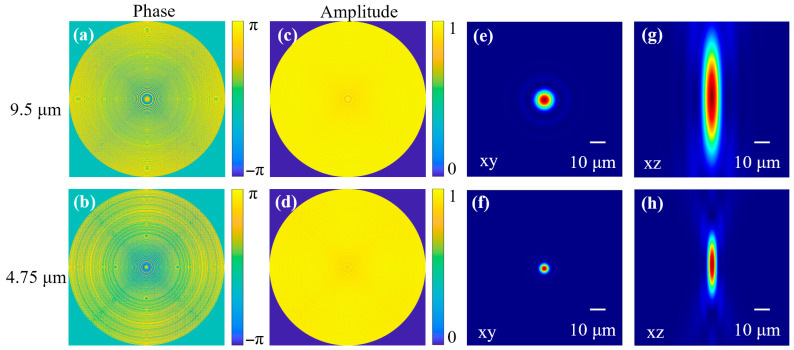
Simulation results of the metalens. (**a**,**b**) show phase distributions of the metalens under incident wavelengths of 9.5 μm and 4.75 μm; (**c**,**d**) show amplitude distributions of the metalens under incident wavelengths of 9.5 μm and 4.75 μm; (**e**,**f**) show the optical intensity distributions at the focal plane under incident wavelengths of 9.5 μm and 4.75 μm; (**g**,**h**) depict the optical intensity profiles measured along the optical axis.

Figure 4a and Figure 4b illustrate the phase distributions of the proposed dual-wavelength metalens under incident wavelengths of 9.5 μm and 4.75 μm, respectively. Both phase profiles exhibit rotational symmetry, a characteristic ensured by the inverse design methodology. Figure 4c,d present the corresponding amplitude distributions, demonstrating average transmission of the selected unit cells is 0.85 at 9.5 μm and 0.67 at 4.75 μm. The far-field optical intensity distribution can be simulated by combining the phase and amplitude profiles of the metalens, as described by the Fresnel–Kirchhoff diffraction equation [38].

Figure 4e and Figure 4f present the optical intensity distributions at the focal plane under incident plane waves of 9.5 μm and 4.75 μm, respectively. The focal spots exhibit full widths at half maximum (FWHM) of 12 μm and 6 μm, corresponding to optical focusing efficiencies of 79% and 60% for incident wavelengths of 9.5 μm and 4.75 μm, under a numerical aperture (NA) of 0.51. The optical intensity distributions along the propagation axis are depicted in Figure 4g,h. The focal lengths corresponding to the 9.5 μm and 4.75 μm incidences are 10 mm. These simulation results substantiate the effectiveness of the proposed inverse design strategy in concurrently optimizing optical performance across both incident wavelengths.

## 4. Fabrication and Experimental Characterization of the Dual-Wavelength Metalens

**Figure 5:**
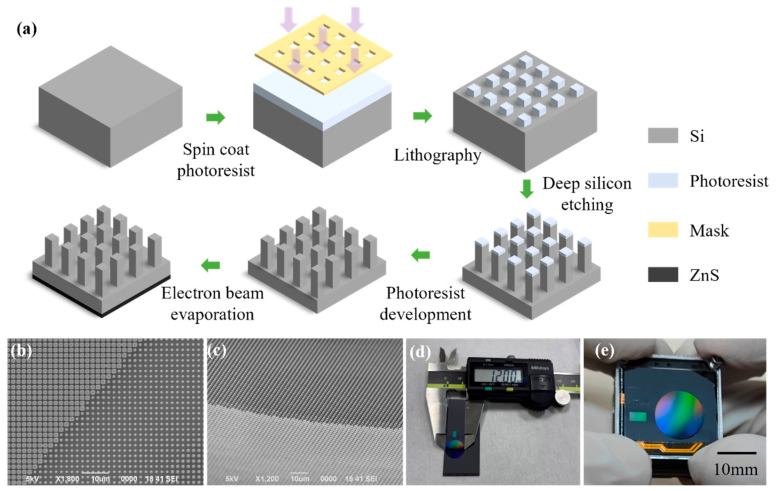
The fabrication processes and Photograph of the sample. (**a**) the fabrication processes of the metalens; (**b**,**c**) the SEM graphs; (**d**,**e**) the photographs of metalens and the thermal imaging system with the size of 26 × 26 × 18 mm^3^ and a total weight of 19 g.

The fabrication of the metalens samples is carried out using a one-step silicon-based lithographic process, which includes: photolithography, deep silicon etching, and electron-beam evaporation of an antireflection coating. The detailed fabrication process is illustrated in Figure 5a. Following standard cleaning procedures, a surface pretreatment step is applied to enhance the adhesion between the silicon wafer and photoresist. The photoresist is then spin-coated onto the wafer, followed by photolithographic exposure using a lithography system capable of achieving the minimum feature size required for the designed unit cell. After exposure, a development process transfers the photomask pattern onto the photoresist layer. Subsequently, deep silicon etching is performed, during which the silicon regions exposed to the etching gases are selectively removed, where the regions protected by the photoresist remain intact, thereby transferring the pattern from the photoresist to the silicon substrate. After etching, the residual photoresist is removed through a chemical cleaning process.

Figure 5b,c present the top-view and side-view scanning electron microscopy (SEM) images of the fabricated metalens, confirming that the structures exhibit well-defined geometries without noticeable defects and that the pillar heights remain within acceptable fabrication tolerances. Figure 5d and Figure 5e display the photograph of the metalens and the integrated thermal imaging system incorporating the metalens, respectively. The fabricated metalens has a diameter of 12 mm, while the thermal imaging system possesses dimensions of 26 × 26 × 18 mm^3^ and a total weight of 19 g.

**Figure 6:**
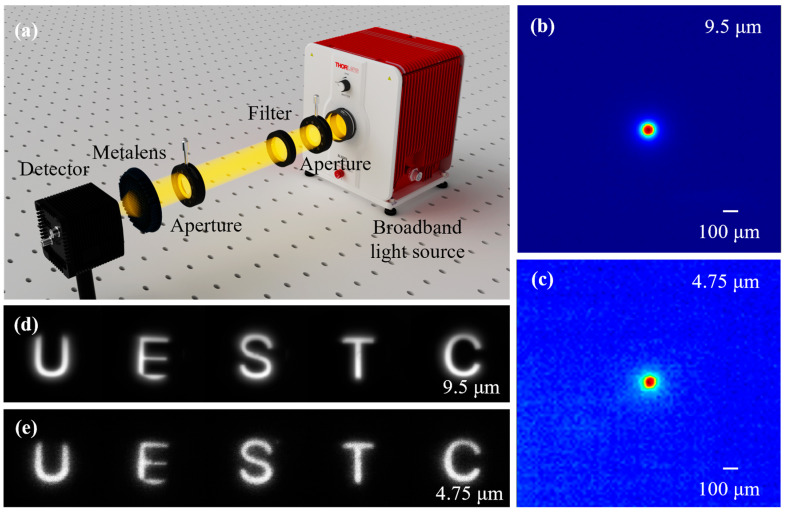
Experimental characterization of the metalens. (**a**) Schematic of the experiment setup. The bandpass filter is placed in front of the metalens to adjust the incident wavelength. The light intensity distribution in the xy plane at different z positions is measured by adjusting the distance between the metalens and the detector plane. the circular aperture with a diameter of 8.4 mm used for imaging is positioned 1000 mm in front of the metalens; (**b**,**c**) Imaged circular aperture at 9.5 μm and 4.75 μm, respectively. The image size of the circular aperture is 85 μm and the image distances for these two wavelengths are 10.1 mm and 10.2 mm; (**d**,**e**) thermal images of UESTC at 9.5 μm and 4.75 μm, respectively.

The experimental characterization setup is schematically illustrated in Figure 6a. Broadband radiation emitted from a silicon nitride source (SLS303) first passes through an aperture and subsequently through narrowband filters corresponding to the two target wavelengths. The filtered beams are then limited by a secondary aperture prior to illuminating the metalens. The output light produced by the metalens is ultimately captured by a microbolometer detector (WinCamD-IR-BB beam profiling camera).

The corresponding optical intensity distributions on the image plane are shown in Figure 6b,c. The measured pattern corresponds to the image of a circular aperture with a diameter of 8 mm, positioned 1 m in front of the metalens. Under illumination at wavelengths of 9.5 μm and 4.75 μm, the measured image distances are 10.1 mm and 10.2 mm, respectively, corresponding to a focal length variation of merely 1%, which shows good agreement with the simulation results. Furthermore, the measured image size of the circular aperture is approximately 85 μm (with a detector pixel size of 17 μm), closely matching the ideal size of 80 μm calculated based on the object-image ratio.

Figure 6d and Figure 6e present the imaging results of the dual-wavelength metalens under illumination at incident wavelengths of 9.5 μm and 4.75 μm, respectively. In both cases, the imaging object is defined by a homemade transparent target. During the experiment, the central wavelength of the bandpass filter was alternated between 9.5 μm and 4.75 μm. The thermal images obtained exhibit high quality. These results demonstrate that the dual-wavelength metalens enables effective thermal imaging in both the LWIR and MWIR spectral regimes within a compact optical system.

## 5. Discussion

In this study, we present an inverse designed metalens capable of dual-wavelength operation at 9.5 μm and 4.75 μm, fabricated via a one-step silicon etching process. The inverse design methodology enables simultaneous optimization of focusing performance at both wavelengths. By explicitly accounting for the contribution of each unit cell to the global lens response, the framework mitigates performance discrepancies and ensures balanced focusing quality across the two operating wavelengths.

Experimental characterization reveals a focal length of 10.048 mm, in close agreement with numerical simulations. Furthermore, we demonstrate a compact dual-wavelength thermal imaging system incorporating the proposed metalens, with dimensions of 26 × 26 × 18 mm^3^ and a total weight of 19 g. These results validate the effectiveness of the inverse design strategy for realizing dual-wavelength metasurfaces.

Despite its advantages, the approach remains constrained by the unit cell database when extending the operational bandwidth. As illustrated in Figure 3, the ideal phase profiles of the metalens are inherently wavelength-dependent, dictated by its planar geometry. In this work, performance enhancement was achieved through modulated phase profiles and unit cell selection guided by the inverse design framework. Nevertheless, extending the design to broadband applications remains challenging. Simulations (Figure 4) indicate absolute efficiencies of 79% and 60% at 9.5 μm and 4.75 μm, respectively, with limitations arising from available unit cell configurations. Table 2 compares the performance parameters of other LWIR and MWIR metalenses. The results show that, with a relatively simple fabrication process, our metalens achieves high focusing efficiency and a large numerical aperture for cross-band focusing and imaging.

Overall, this work establishes a robust and scalable inverse design paradigm for dual-wavelength metalenses. The proposed frame can be readily adapted to alternative wavelength combinations and functional requirements, thereby paving the way for compact, integrated, and multifunctional infrared optical systems with promising applications in multispectral imaging, sensing, and detection.

## 6. Materials and Methods

Fabrication. Firstly, the silicon wafer surface is cleaned successively with acetone, anhydrous ethanol and deionized water, and then dried. Next, it is pre-treated with hexamethyldisilazane (HMDS) for 12 min. AR80 photoresist is applied: the silicon wafer rotates at 600 rpm for 30 s, then rotates at 4000 rpm for 60 s, forming a photoresist layer approximately 800 nm thick. Soft baking is carried out at 95 °C for 90 s. A Nikon I12 ( made in Nikon, Tokyo, Japan) is used for lithography, with an exposure time of 270 ms, followed by exposure and baking at 110 °C for 60 s. After 25 °C, the sample is developed with NMD-3 developer (2.38%) for 50 s. Then, deep silicon etching is performed with a substrate power of 50 W, SF6 flow rate of 120 sccm, and C4F8 flow rate of 80 sccm. In each etching cycle, the passivation and etching times are 1.2 s and 1.3 s respectively, and the chamber pressures are 16 and 18 mTorr. Finally, the photoresist is removed with a mixture of concentrated sulfuric acid and hydrogen peroxide, resulting in the finished sample. Subsequently, a 0.95 μm thick ZnS layer is deposited on the back of the metal lens using an electron beam evaporation system, with an internal pressure of 10^−4^ Torr. The anti-reflective coating increases the light transmittance for 9.5 μm light by 17%, and has almost no improvement on the transmittance for 4.75 μm light.

Experiment. The broadband light source used in the experiment is a stabilized free-space source SLS303 (made in Thorlabs, Newton, New Jersey, USA), providing a spectral range from 550 nm to 15 μm. The narrowband filters employed are Thorlabs FB4750-500 and FB9500-500. The detector is a WinCamD-IR-BB beam profiling camera (made in 1675 Market Street, Redding, CA 96001 USA), capable of operating over the 2–16 μm wavelength range.

## Figures and Tables

**Table 1:** Wrapped phase and phase difference of 9.5 μm and 4.75 μm with different *W*.

*W* (μm)	Wrapped Phase 9.5 μm (rad)	Wrapped Phase 4.75 μm (rad)	Phase Difference (rad)
0.8	−0.5569	2.6049	3.1618
0.9	−0.8770	−0.5582	0.3188
1.0	−1.2855	2.2822	3.5678
1.1	−1.8216	−0.5830	1.2386
1.2	−2.5173	−2.4869	0.0304

**Table 2:** Performance comparison of dual-wavelength metalens.

	Simulation Efficiency (%)	Diameter (mm)	NA	Bandwidth	Preparation Complexity	Reference
1	31.0	0.4	0.32	Single band	Low	[39]
2	15.2	31.8	0.45	Single band	Low	[40]
3	73.0	1	0.60	Single band	Medium	[41]
4	70.0	0.37	0.42	Single band	Low	[42]
5	<35	38	0.71	Dual band	High	[35]
	69.5	12	0.51	Dual wavelength	Low	Our work

## Data Availability

The original contributions presented in this study are included in the article. Further inquiries can be directed to the corresponding author.
